# Tryptanthrin Inhibits Angiogenesis by Targeting the VEGFR2-Mediated ERK1/2 Signalling Pathway

**DOI:** 10.1371/journal.pone.0082294

**Published:** 2013-12-16

**Authors:** Xuemei Liao, Xuelin Zhou, Nai-ki Mak, Kwok-nam Leung

**Affiliations:** 1 Biochemistry Programme, School of Life Sciences, The Chinese University of Hong Kong, Shatin, HKSAR, China; 2 School of Biomedical Sciences, The Chinese University of Hong Kong, Shatin, HKSAR, China; 3 Department of Biology, Hong Kong Baptist University, Kowloon Tong, HKSAR, China; Medical College of Wisconsin, United States of America

## Abstract

Angiogenesis is a key step for tumour growth and metastasis, and anti-angiogenesis has been proposed as an important strategy for cancer therapy. Tryptanthrin is a weakly basic alkaloid isolated from the dried roots of medicinal indigo plants and has been shown to possess anti-tumour activities on various cancer cell types. This study aims to investigate the *in vitro* and *in vivo* anti-angiogenic activities of tryptanthrin and to unravel its underlying molecular action mechanisms. Our results show that tryptanthrin inhibited the *in vitro* proliferation, migration, and tube formation of the human microvascular endothelial cells (HMEC-1) in a concentration-dependent manner and significantly suppressed angiogenesis in Matrigel plugs in mice. Mechanistic studies indicated that tryptanthrin reduced the expression of several pro-angiogenic factors (Ang-1, PDGFB and MMP2). Tryptanthrin was also found to suppress the VEGFR2-mediated ERK1/2 signalling pathway in HMEC-1 cells and molecular docking simulation indicated that tryptanthrin could bound to the ATP-binding site of VEGFR2. Collectively, the present study demonstrated that tryptanthrin exhibited both *in vitro* and *in vivo* anti-angiogenic activities by targeting the VEGFR2-mediated ERK1/2 signalling pathway and might have therapeutic potential for the treatment of angiogenesis-related diseases.

## Introduction

Angiogenesis, the formation of new blood vessels from pre-existing vascular network, plays an important role in the tumour growth, invasion and metastasis [1], [2]. During tumour growth, tumour cells secrete pro-angiogenic proteins such as vascular endothelial growth factor (VEGF), angiopoietins (Ang), platelet-derived growth factor (PDGF) and matrix metalloproteinases (MMP) to stimulate endothelial cell proliferation, migration and vascular tube formation [3], [4]. VEGF was proven to be one of the key regulators in the process of angiogenesis [5]. Vascular endothelial growth factor receptor 2 (VEGFR2) is the primary receptor of VEGF and the major mediator of VEGF-induced pro-angiogenesis signalling in endothelial cells [6], [7]. Binding of VEGF to VEGFR2 leads to dimerization of receptors, activation of tyrosine kinase, trans-autophosphorylation of specific tyrosine residues within the cytoplasmic domain and initiation of intracellular signalling cascades including activation of extracellular signal-regulated kinase (ERK), phosphoinositide 3-kinase - protein kinase B (PI3K-AKT), c-Jun N-terminal kinase (JNK) and p38 mitogen-activated protein kinase (p38 MAPK) [7]–[12]. Tumour angiogenesis is an important control point in the progression of cancer and its inhibition is emerging as a potentially valuable new approach to cancer therapy [1], [7], [13]. Administration of Bevacizumab (Avastin), a humanized VEGF-A antibody, significantly improved the survival rates in advanced colorectal cancer patients [14], [15]. Multiple tyrosine kinase inhibitors of VEGFR and other growth factor receptors, such as Sunitinib and Sorafenib, have been successfully used in the clinic to treat renal carcinoma [13], [16].

In recent years a number of anti-cancer compounds with anti-angiogenic activity have been derived from natural products or structurally modified natural compounds such as Philinopside A [17], triptolide [18] plumbagin [19] and pristimerin [20]. Tryptanthrin (12-dihydro-6, 12-dioxoindolo-(2, 1-b)-quinazoline) is a low molecular weight compound isolated from the dried roots of medicinal indigo plants (Banlangen). Extensive studies reported that tryptanthrin possesses multiple biological and pharmacological activities including anti-microbial [21], anti-inflammatory [22], anti-allergic [23] and anti-tumour activity [24]–[26]. In addition, tryptanthrin was found to reverse doxorubicin resistance in the breast cancer cell line MCF-7 by suppressing the expression of multi-drug resistance (MDR) 1 gene [25]. More recently, we found that tryptanthrin suppressed the growth and induced neuronal differentiation of the human neuroblastoma LA-N-1 cells [27]. However, the effects of tryptanthrin on angiogenesis and the underlying molecular mechanisms have not yet been investigated.

In this study, the anti-angiogenic effects of tryptanthrin were evaluated both *in vitro* and *in vivo*. Mechanistic study further indicated that tryptanthrin significantly inhibited the expression of several pro-angiogenic factors such as Ang-1, PDGFB and MMP2, suppressed the phosphorylation of VEGFR2 and blocked the VEGFR2-mediated ERK1/2 signalling pathway.

## Materials and Methods

### Cell Culture and Reagents

**Figure 1:**
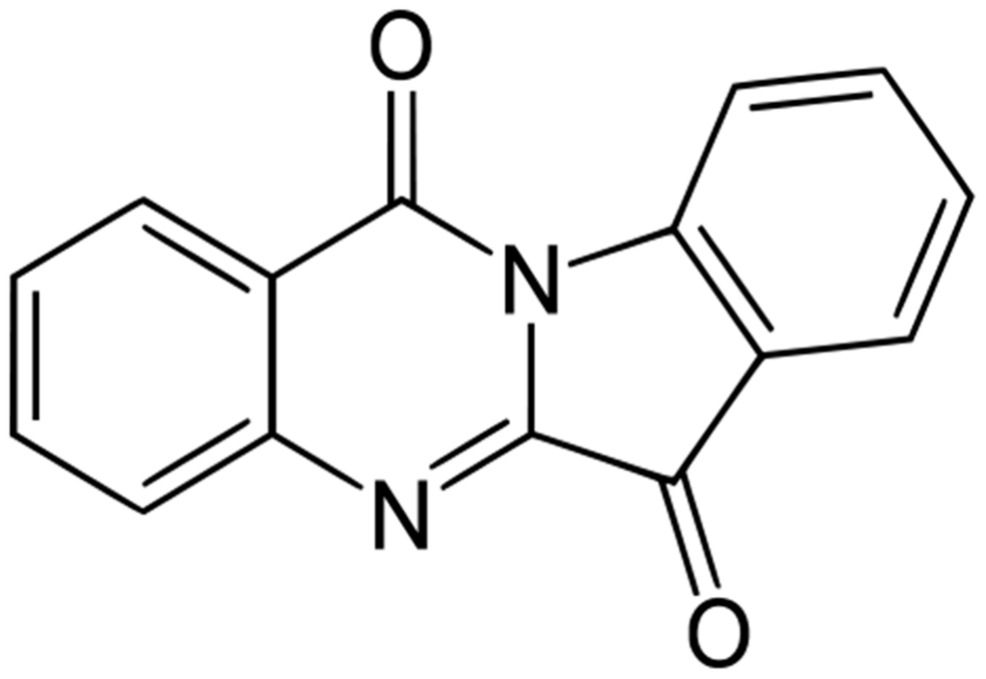
Chemical structure of tryptanthrin.

The human microvascular endothelial cell line HMEC-1 was purchased from the American Type Culture Collection (ATCC, USA) and maintained in MCDB131 medium (GIBCO, USA) supplemented with 10% fetal bovine serum (FBS) (GIBCO), 100 units/mL penicillin–streptomycin (GIBCO), 2 mM glutamine (GIBCO), 1 mg/mL hydrocortisone (Sigma, USA) and 10 ng/mL epidermal growth factor (EGF) (Sigma, USA) in a humidified incubator with 5% CO_2_ at 37°C. Tryptanthrin (Figure 1) used in this study was purchased from Alexis Biochemicals (USA). The powdered compound (with molecular weight 248.2 and >98% purity as determined by ^1^H-NMR and HPLC) was dissolved in sterile, cell-cultured tested dimethyl sulfoxide (DMSO) (Sigma, USA) to give 10 mM stock solutions which were stored in the dark at −20°C until use. All other chemicals were purchased from Sigma unless otherwise stated.

### Proliferation and Cytotoxicity Assays

The effects of tryptanthrin on cell proliferation and cytotoxicity were measured using the CyQUANT® NF Cell Proliferation Assay (Invitrogen, USA) and CytoTox 96® Non-Radioactive Cytotoxicity Assay (Invitrogen, USA), respectively. Briefly, HMEC-1 cells were seeded in 96-well plates and cultured overnight followed by treatment with different concentrations of tryptanthrin or solvent control. After treatment for the indicated periods of time, cell proliferation was measured using the CyQUANT® NF Cell Proliferation Assay Kit and the fluorescence signals were recorded by a fluorescence plate reader (TECAN Polarion, USA). Cell cytotoxicity was measured using the CytoTox 96® Non-Radioactive Cytotoxicity Assay Kit and the optical density of the color product was recorded by a BENCHMARK microplate reader (Bio-Rad Laboratories, USA).

### Wound Healing Assay

The HMEC-1 cells were allowed to grow to full confluence in 6-well plates, then wounded by scratching with a 0.1 mL pipette tip, and the wells were washed with phosphate-buffered saline (PBS) to remove the detached cells. Cells were then incubated with various concentrations of tryptanthrin or solvent control for 8 h. Migration ability was evaluated by measuring the gap widths narrowed down by HMEC-1 cells’ movement. Four images in each well were taken under Nikon Multi-zoom AZ-C2 Macro Confocal System (Nikon, Japan), and the gap width of each image was measured and averaged.

### Tube Formation Assay

The tube formation assay was performed as described by Chan *et al*. with slight modifications [28]. Briefly, each well of the 24-well plates were coated with 100 µL Matrigel basement membrane matrix (BD Bioscience, USA) and allowed to polymerize at 37°C for 30 min. HMEC-1 cells were then seeded into the wells of 24-well plates with the addition of various concentrations of tryptanthrin or solvent control. After 16 h, the network of tubes formed was observed under Nikon Multi-zoom AZ-C2 Macro Confocal System, and photographed in 3 random regions. The number of branch points of individual polygons of the capillary network was counted.

### *In vivo* Matrigel Plug Assay

All animal care and experimental procedures were compliant with the guidelines of the Animal Experimentation Ethics Committee of The Chinese University of Hong Kong and approval to conduct the animal experiments had been obtained from this committee (Animal Experimentation Ethics Approval Ref. No. 12/064/GRF and 468712). Male BALB/c mice (6 weeks old) were supplied and maintained by Laboratory Animal Service Center, The Chinese University of Hong Kong. The Matrigel plug assay in BALB/c mice was performed as described previously [29]. Briefly, tryptanthrin or solvent control in 50 µL PBS was mixed with 450 µL Matrigel (BD Bioscience, USA) containing heparin (40 units/mL) and recombinant mouse VEGF-A (100 ng/mL). Prepared Matrigel was then injected subcutaneously into the flanks of 6-week-old BALB/c male mice. After 7 days, the Matrigel plugs were removed and photographed. The hemoglobin content of the Matrigel plugs was quantified using QuantiChrom™ Hemoglobin Assay Kit (BioAssay Systems, USA).

### Quantitative Real-time PCR

Total RNA from HMEC-1 cells was extracted by Trizol reagent (Invitrogen, USA) according to the manufacturer’s protocol. The first-strand cDNA was generated with random primer (Invitrogen, USA) using the M–MLV reverse transcription kit (Invitrogen, USA). Quantitative real-time PCR analysis was performed with SYBR premix Ex Taq kit (TaKaRa, China) using the ABI-7500 Real-Time PCR System (Applied Biosystems, USA). Relative gene expression was normalized to β-actin levels. The sequences of primers used are listed in Table 1.

**Table 1:** Sequences of primers used for RT-PCR are based on human genes and shown from 5′ to 3′.

β-actin-Forward strand	CAGGAGATGGCCACTGCCGCA
β-actin-Reverse strand	CTCCTTCTGCATCCTGTCAGCA
Ang-1-Forward strand	CCAGTACAACACAAACGCTCT
Ang-1-Reverse strand	TCTCCGACTTCATGTTTTCCAC
Ang-2-Forward strand	CTCGAATACGATGACTCGGTG
Ang-2-Reverse strand	TCATTAGCCACTGAGTGTTGTTT
PDGFA-Forward strand	GCAAGACCAGGACGGTCATTT
PDGFA-Reverse strand	GGCACTTGACACTGCTCGT
PDGFB-Forward strand	AGCCTGTGGCTTGGAGTG
PDGFB-Reverse strand	ACCCACCTAGAAGGGCAGTT
MMP2-Forward strand	CCCACTGCGGTTTTCTCGAAT
MMP2-Reverse strand	CAAAGGGGTATCCATCGCCAT
MMP9-Forward strand	TGTACCGCTATGGTTACACTCG
MMP9- Reverse strand	GGCAGGGACAGTTGCTTCT

### Phospho-VEGFR2 Sandwich ELISA Assay

Endogenous level of phospho-VEGFR2 (Tyr1175) protein was measured using the PathScan® Phospho-VEGFR2 (Tyr1175) Sandwich ELISA Kit (Cell Signaling Technology, USA). Briefly, cell pellets were collected at the indicated time points and total proteins were extracted by the cell lysis buffer. Protein concentration was measured by Bradford reagent (Sigma, USA). The protein samples were diluted to the same concentration and 100 µL of each diluted cell lysate was added to the mouse anti-VEGFR-2 antibody-coated microwells and allowed to incubate overnight. Then the microwells were incubated with rabbit anti-p-VEGFR2 (Y1175) detection antibody, followed by horseradish peroxidase (HRP)-linked secondary antibody, and then developed with 3,3′,5,5′-tetramethylbenzidine (TMB) substrate. Absorbance was read at 450 nm after adding the stop solution and the results were recorded by a BENCHMARK microplate reader.

### Western Blot Analysis

Cell pellets were collected at the indicated time points and total proteins were extracted by the cell lysis buffer. Protein concentration was measured by Bradford reagent. Protein samples were resolved on 10% polyacrylamide gels, transferred to polyvinylidene fluoride (PVDF) membrane and blocked with 5% non-fat dairy milk in Tris-buffered saline (20 mM Tris, 150 mM NaCl, pH 7.4) with 0.05% Tween-20. Membranes were incubated with the following primary antibodies: mouse anti-human phospho-ERK1/2 (p-ERK1/2) antibody (Cell Signaling Technology, USA), rabbit anti-human ERK1/2, p-AKT, AKT and JNK antibodies (Cell Signaling Technology, USA), rabbit anti-human p-p38 and p38 antibodies (Santa Cruz Biotechnology, USA), mouse anti-human p-JNK (Santa Cruz Biotechnology, USA), mouse anti-human β-actin antibody (Sigma, USA), followed by HRP-conjugated secondary antibody (GE Healthcare Limited, UK) and developed with ECL reagent (Santa Cruz Biotechnology). The protein band intensity was quantified using Quantity One software (Bio-Rad Laboratories, USA). The results were expressed as relative protein levels compared to the relative total protein ± SD of three independent experiments.

### Molecular Docking

Interaction of tryptanthrin with the ATP-binding site in the kinase-insert domain-containing region (KDR) of VEGFR2 was measured by molecular docking analysis with AutoDock Vina v.1.0.2 [30]. The crystal structure of VEGFR2 (PDB code 1YWN) was obtained from the Protein Data Bank. The docking parameters were set using default values with the grid box (size: 20 Å×20 Å×20 Å) encompassing the ATP-binding site [31]. The binding modes of tryptanthrin with the lowest binding free energy to the ATP-binding site were chosen for subsequent confirmation of docking conformation. The simulation data were illustrated by PyMOL v.1.3 (Schrödinger, OR, USA) and Discovery Studio Visualizer (Accelrys, CA, USA).

### Statistical Analysis

Assays done in triplicate were performed at least three times and the data are presented as mean ± SD. Student’s *t*-test was used to determine the significant difference between the drug-treated group and the control group. P<0.05 is regarded as statistically significant different.

## Results

### Tryptanthrin Inhibited the Proliferation of Human Microvascular Endothelial Cells

**Figure 2:**
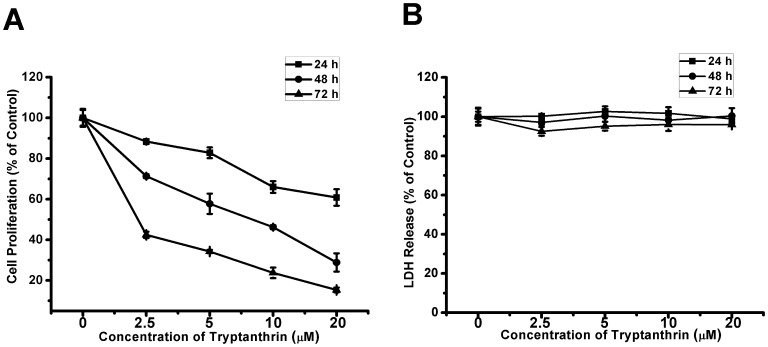
Anti-proliferative effect of tryptanthrin on human microvascular endothelial cells. HMEC-1 cells (3×10^3^ cells/well) were cultured overnight in 96-well plates and treated with solvent control (0.1% DMSO) or various concentrations of tryptanthrin for 24 h, 48 h or 72 h. After treatment, (A) the relative cell proliferation was measured using the CyQUANT® NF Cell Proliferation Assay Kit and (B) LDH release was determined using the CytoTox 96® Non-Radioactive Cytotoxicity Assay Kit. The relative cell proliferation and LDH release for the solvent control treatment was set as 100%. Data are expressed as mean ± SD.

To study the anti-angiogenic activity of tryptanthrin *in vitro,* the effect of tryptanthrin on the proliferation of the human microvascular endothelial HMEC-1 cells was first examined. HMEC-1 cells were incubated with increasing concentrations of tryptanthrin for 24 h, 48 h and 72 h. Cell proliferation was monitored by using the CyQUANT® NF cell proliferation assay. As shown in Figure 2A, tryptanthrin inhibited the proliferation of HMEC-1 cells in a concentration- and time-dependent manner. However, tryptanthrin exhibited no significant cytotoxicity on HMEC-1 cells as measured by the LDH release assay (Figure 2B), indicating that the anti-proliferative effect was not due to the direct cytotoxic action of the tryptanthrin on the HMEC-1 cells.

### Tryptanthrin Suppressed Migration and Tube Formation of Human Microvascular Endothelial Cells

**Figure 3:**
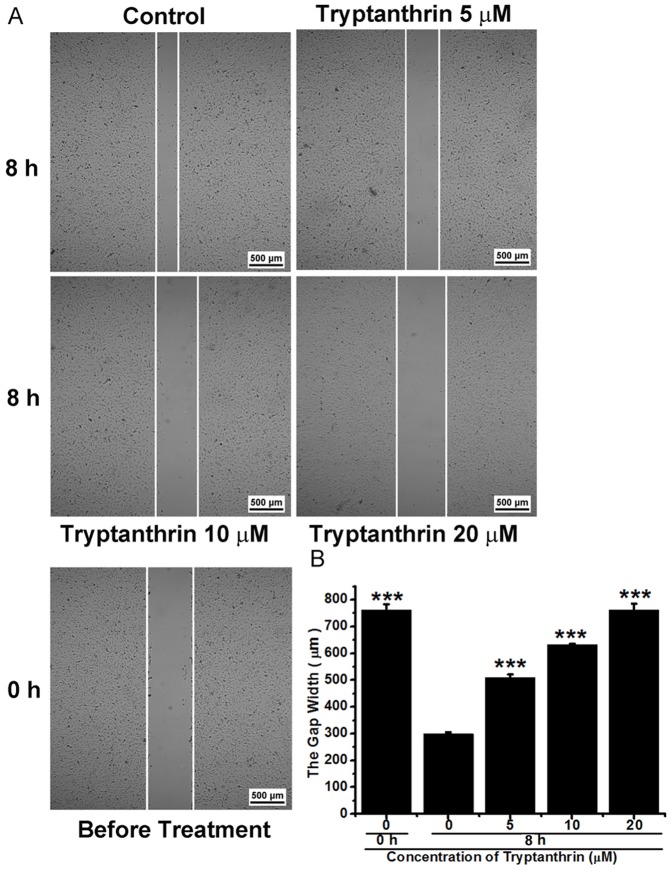
Tryptanthrin inhibited the migration of human microvascular endothelial cells. HMEC-1 cells (5×10^5^cells/well) were wounded by scratching with a 0.1 mL pipette tip and were incubated with solvent control (0.1% DMSO) or various concentrations of tryptanthrin for 8 h. (A) Representative appearance of the HMEC-1 cells at 0 h and 8 h. (B) Migration ability was evaluated by measuring the gap widths narrowed down by HMEC-1cells’movement. Data are expressed as mean ± SD. *** p<0.001.

**Figure 4:**
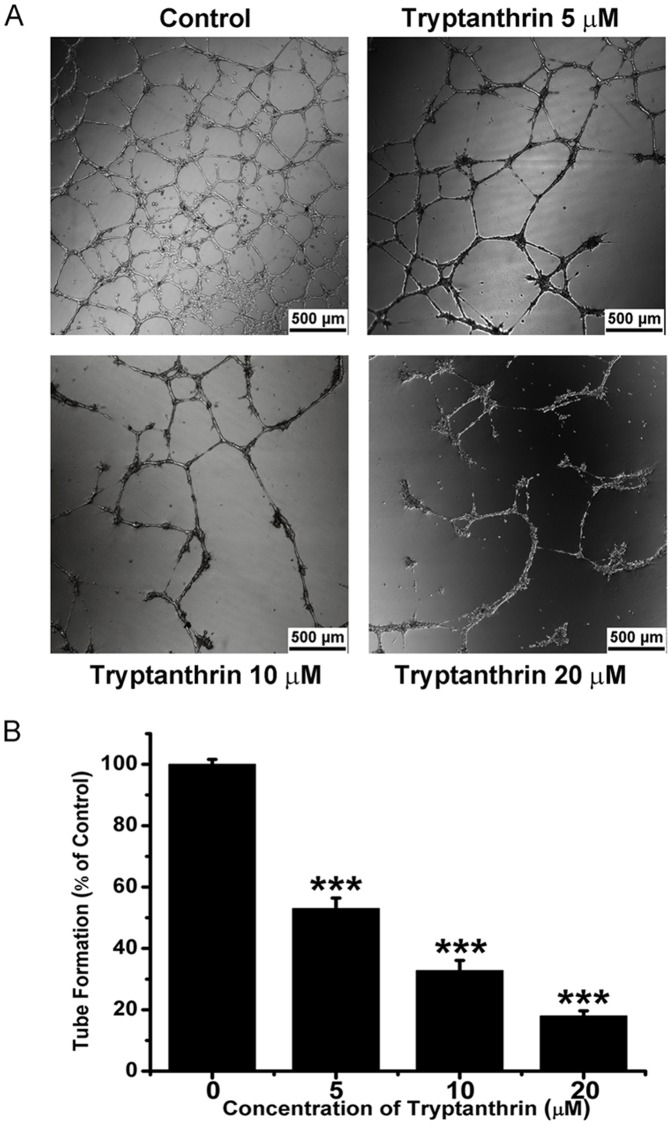
Tryptanthrin inhibited the capillary-like tube formation of human microvascular endothelial cells. HMEC-1 cells (1.5×10^5^cells/well) were seeded onto Matrigel-coated 24-well plates and incubated with solvent control (0.1% DMSO) or various concentrations of tryptanthrin for 16 h. (A) Representative appearance of HMEC-1 tube formation. (B) The tube formation was quantified by counting the number of branch points of the capillary network. Data are expressed as mean ± SD. *** p<0.001.

Endothelial cell migration and tube formation are early events and necessary steps involved in tumour angiogenesis [32], [33]. In the present study, the wound healing assay was used to determine the effect of tryptanthrin on HMEC-1 cell migration. As shown in Figure 3, treatment of HMEC-1 cells with tryptanthrin for 8 h inhibited the closure of the wound gap in a concentration-dependent manner, indicating that tryptanthrin strongly inhibited the migration of HMEC-1 cells. Moreover, endothelial cells are able to form capillary-like structure when seeded on basement membrane matrix such as Matrigel [28]. In the tube formation assay, it was found that the amount and the integrity of tubular capillary-like network on Matrigel were significantly inhibited by tryptanthrin at 16 h (Figure 4). Taken together, these results suggested that tryptanthrin could inhibit angiogenesis *in vitro*.

### Tryptanthrin Blocked Angiogenesis in the *in vivo* Matrigel Plug Model

**Figure 5:**
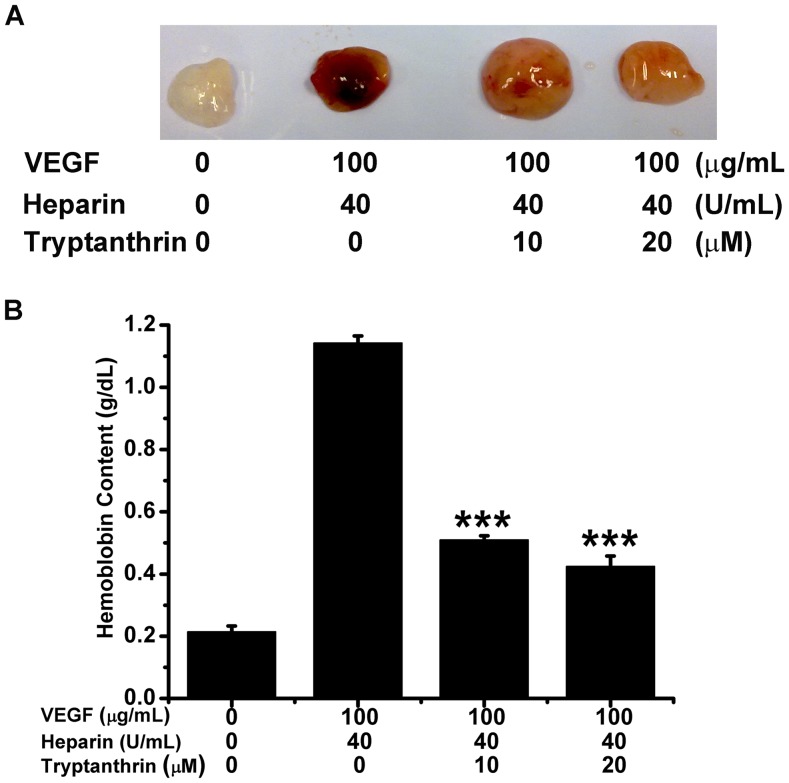
Tryptanthrin suppressed the Matrigel plug *in vivo* angiogenesis. Matrigel mixed with VEGF and heparin or tryptanthrin was injected into the flanks of 6-week-old BALB/c mice (five mice per group). Seven days later, the Matrigel plugs were removed for analysis. (A) Representative appearance of Matrigel plugs. (B) Hemoglobin content of Matrigel plugs from groups of mice was quantified by using QuantiChrom™ Hemoglobin Assay Kit. Data are expressed as mean ± SD. *** p<0.001.

To further verify the anti-angiogenic effect of tryptanthrin, the *in vivo* Matrigel plug assay was used [29]. Matrigel containing recombinant mouse VEGF and heparin with or without tryptanthrin (10 and 20 µM) was injected into the flanks of BALB/c mice. After 7 days, the mice were sacrificed and the Matrigel plugs were removed. The plugs containing VEGF and heparin exhibited red color, indicating that occurrence of angiogenesis (Figure 5A). In the presence of tryptanthrin, the plugs were of light red or pale yellow color, indicating there was a decrease in angiogenesis (Figure 5A). The extent of angiogenesis was then quantified by measuring the hemoglobin content in the plugs. As shown in Figure 5B, tryptanthrin significantly reduced the hemoglobin concentration in the plugs induced by VEGF and heparin. This observed *in vivo* anti-angiogenic activity is consistent with the *in vitro* anti-angiogenic effect of tryptanthrin.

### Tryptanthrin Reduced the Expression of Angiogenic Genes in Human Microvascular Endothelial Cells

**Figure 6:**
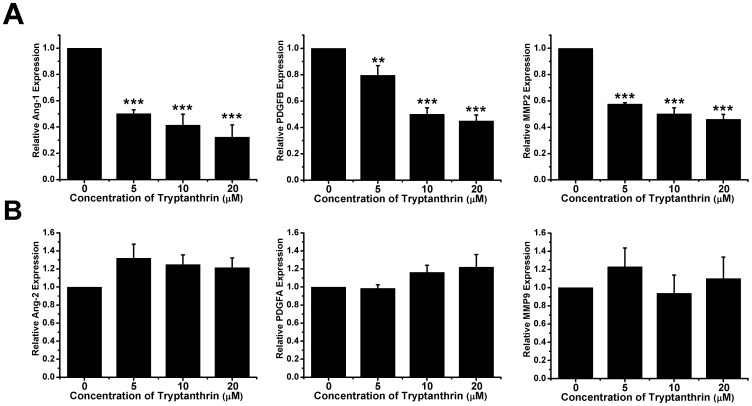
Effect of tryptanthrin on the expression of angiogenic factors in human microvascular endothelial cells. (A-B) HMEC-1 cells were treated with solvent control (0.1% DMSO) or various concentrations of tryptanthrin for 24 h. mRNA expression levels of Ang-1, Ang-2, PDGFA, PDGFB, MMP2 and MMP9 were measured using real-time PCR. Results represent mean ± SD. ** p<0.01; *** p<0.001.

Angiogenic factors play an important role in the regulation of angiogenesis and tumour progression. Since tryptanthrin exerted both *in vitro* and *in vivo* anti-angiogenic activities, we sought to determine the expression of several angiogenic factors including angiopoietin-1 (Ang-1), Ang-2, platelet-derived growth factor A (PDGFA) and PDGFB, matrix metalloproteinase 2 (MMP2) and MMP9. Results in Figure 6A showed that tryptanthrin decreased the expression of Ang-1, PDGFB and MMP2 in a concentration-dependent manner. However, significant suppressive effect of tryptanthrin on the expression of Ang-2, PDGFA and MMP9 was not observed (Figure 6B). These findings suggest that tryptanthrin might inhibit angiogenesis partly through transcriptional suppression of several specific angiogenic gene expressions.

### Tryptanthrin Attenuated VEGFR2-mediated ERK1/2 Pathway in Human Microvascular Endothelial Cells

**Figure 7:**
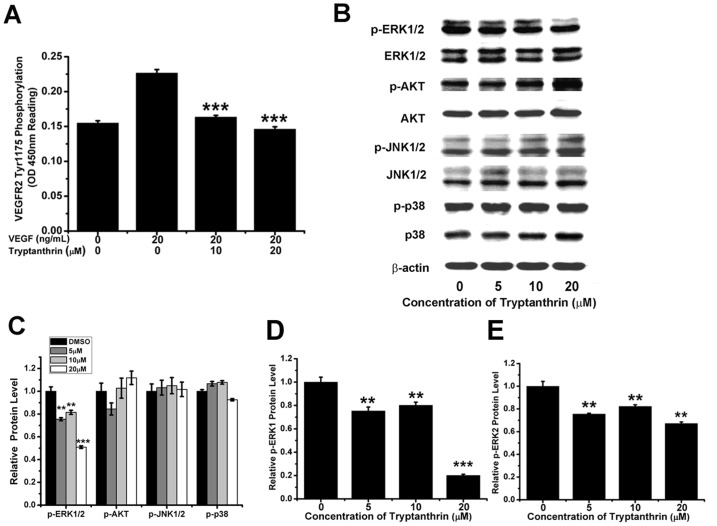
Effect of tryptanthrin on the VEGFR2 signalling pathways in human microvascular endothelial cells. (A) The phosphorylation activity of VEGFR2 in the Tyr1175 phosphorylation site was measured after treatment with tryptanthrin for 4 h using the PathScan® Phospho-VEGFR-2 (Tyr1175) Sandwich ELISA Kit. (B) Protein levels of phosphorylated and unphosphorylated ERK, AKT, JNK and p38 in HMEC-1 cells were measured after treatment with various concentrations of tryptanthrin for 4 h using β-actin as an internal control. (C) The protein intensity was quantified using Quantity One software and the relative protein levels of p-ERK1/2, p-AKT, p-JNK1/2 and p-p38 compared to the corresponding total protein levels were determined. (D) The relative protein levels of p-ERK1 after tryptanthrin treatment. (E) The relative protein levels of p-ERK2 after tryptanthrin treatment. Results represent mean ± SD. ** p<0.01; *** p<0.001.

Since VEGFR2 is the major receptor of VEGF that mediates angiogenic activity, and VEGF/VEGFR2 pathway plays a key role in angiogenesis, therefore, the effect of tryptanthrin on VEGF-induced tyrosine phosphorylation of VEGFR2 was first examined. The endogenous level of phospho-VEGFR2 (Tyr1175) protein (the active form of VEGFR2) was measured using the PathScan® Phospho-VEGFR2 (Tyr1175) Sandwich ELISA Kit. As shown in Figure 7A, the level of phospho-VEGFR2 (Tyr1175) in HMEC-1 cells was increased by the exogenously added VEGF. Interestingly, the VEGF-induced increase in the level of phospho-VEGFR2 (Tyr1175) could be blocked by tryptanthrin.

To further determine the effect of tryptanthrin treatment on the VEGFR2-mediated signalling, several key VEGFR2 downstream signalling molecules were studied. Results in Figure 7B and 7C showed that tryptanthrin significantly suppressed the phosphorylation of ERK1/2, while the phosphorylation status of other kinases such as AKT, JNK and p38 were not significantly affected when compared to the corresponding total (phosphorylated and unphosphorylated) protein levels. Further analysis indicated that tryptanthrin exerted a more pronounced effect on suppressing the phosphorylation of ERK1 as compared to ERK2 in HMEC-1 cells (Figure 7D and 7E). This observation clearly indicated that tryptanthrin could block the VEGFR2-mediated ERK1/2 signalling pathway.

Tryptanthrin Bound to the ATP-binding Site of VEGFR2 Kinase Domain.

**Figure 8:**
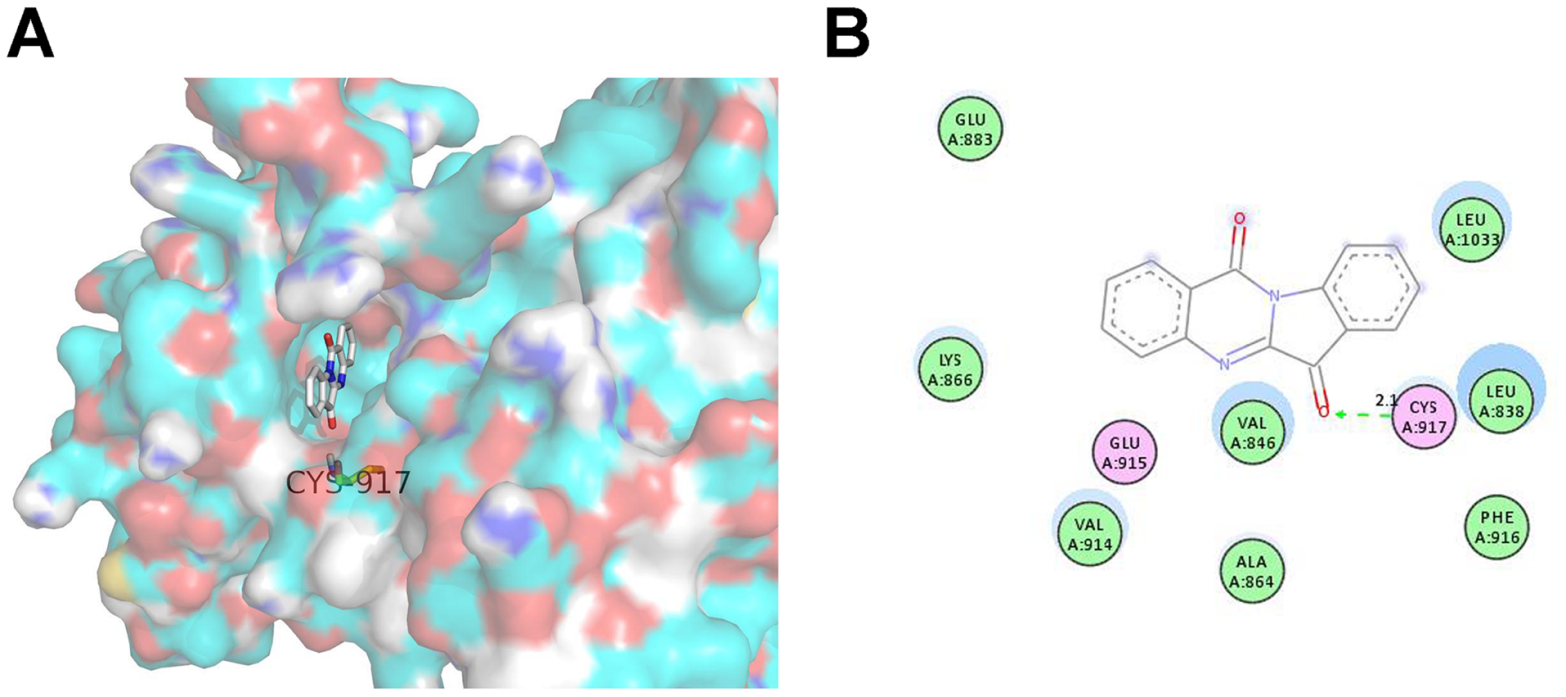
The favourable binding position of tryptanthrin with lowest binding free energy in the ATP-binding site of VEGFR2 (PDB code 1YWN) as analyzed by molecular docking study. (A) The three-dimensional diagram displays the interaction of tryptanthrin (the white stick) to the ATP-binding site of VEGFR2 with the labelled amino acid residue Cys917 which significantly contributed to the binding. (B) The two-dimensional diagram shows the interactions of tryptanthrin to the amino acid residues in the ATP-binding site. Colors of the residues indicate the forms of interactions as follows: van der Waals forces, green; polarity, magenta. Green arrow represents H-bonding with the amino acid main chain.

To further understand how tryptanthrin exerted its anti-angiogenic effects via VEGFR2 and its signalling pathways, the possible binding interaction between tryptanthrin and VEGFR2 kinase domain was analyzed by molecular docking method. As shown in Figure 8A, tryptanthrin could bind to the ATP-binding site of VEGFR2. It was found that tryptanthrin bound to the active cavity of VEGFR2 with the binding energy of −7.8 kcal/mol, which is lower than the binding energy of ATP (−6.4 kcal/mol) (Figure 8A and 8B). In a previous study, the amino acid residues in the active site of VEGFR2, including Cys917 and Glu883, were shown to be responsible for the ligand binding [34]. As shown in Figure 8B, tryptanthrin could interact with the main chain of Cys917 through the H-bonding in the distance of 2.1 Å. It also interacted via Van der Waals forces with Leu838, Val 846, Ala864, Lys866, Glu883, Val914, Phe916 and Leu1033, and via polarity with Glu915 and to a lesser extent with Cys917. These interactions might be responsible for the binding of tryptanthrin to the ATP-binding site of VEGFR2, and hence inhibited the function of VEGFR2.

## Discussion

Tumour angiogenesis is the crucial step in the growth and metastasis of various solid tumours, therefore, anti-angiogenesis has been proposed as an important strategy in cancer therapy. Tryptanthrin, a weakly basic alkaloid isolated from the dried roots of medicinal indigo plants (Banlangen), has been found to possess anti-cancer properties on various cancer cell lines *in vitro* [25]–[27], [35]. However, its inhibitory effect on angiogenesis in endothelial cells and the underlying molecular action mechanisms have not yet been examined. In this study, the anti-angiogenic effect of tryptanthrin was demonstrated for the first time using the human microvascular endothelial HMEC-1 cells. HMEC-1 is an immortalized cell line that are known to retain the morphologic, phenotypic, and functional characteristics of normal human microvascular endothelial cells [36]. The HMEC-1 cells are generally better characterized and more stable as compared to human umbilical vein endothelial cells (HUVEC) which are macrovascular cells with limited life span and possessing various characteristics due to multi-donor origin and their derivation from large vessels [28], [36], [37].

In the process of angiogenesis, endothelial cells proliferate and migrate in response to pro-angiogenic signals (e.g. angiogenic factors), resulting in the formation of capillary sprouts and tubes with a new basement membrane [32], [38]. Our *in vitro* study demonstrated that tryptanthrin inhibited the proliferation of HMEC-1 cells in a concentration- and time-dependent manner whereas it did not pose significant cytotoxicity to HMEC-1 cells under similar conditions. In addition, tryptanthrin significantly interrupted the migration and capillary-like structure formation of endothelial cells. On the other hand, the Matrigel plug assay mimics not only the normal physiological conditions but also reflects many features of tumour angiogenesis *in vivo* and is widely used for the quantitative assessment of neo-angiogenesis [39]. Therefore, in the present study the Matrigel plug assay was used to evaluate the *in vivo* anti-angiogenic effect of tryptanthrin. The results clearly indicated that tryptanthrin treatment could significantly inhibit angiogenesis as judged from the decreased hemoglobin content in the Matrigel plugs in the presence of tryptanthrin. These results, when taken together, strongly suggest that tryptanthrin is an anti-angiogenic agent, both *in vitro* and *in vivo.*

The underlying molecular action mechanism for the anti-angiogenic effect of tryptanthrin was also examined. Angiogenic growth factors are locally released from a growing tumour to stimulate endothelial cell proliferation, migration and extracellular matrix degradation, which are necessary for tumour invasion and new blood vessel formation. Ang-1 (the ligand for TIE-2), a receptor-like tyrosine kinase expressed almost exclusively in endothelial cells, is known to maintain vessel integrity by mediating interactions between the endothelium and surrounding matrix [40]. Ang-2 is a naturally-occurring antagonist of Ang-1 which binds with similar affinity to TIE-2, but disrupts *in vivo* angiogenesis [41]. PDGFA and PDGFB are angiogenic proteins that are involved in the regulation of cell migration and proliferation, and are necessary for the recruitment of pericytes to newly formed blood vessel [42], [43]. On the other hand, MMP2 and MMP9 are involved in the breakdown of the underlying basement membrane of the existing blood vessels which is a crucial step for initiating the formation of new capillaries [44]. Using quantitative RT-PCR, our results demonstrated that tryptanthrin could selectively down-regulate the expression of Ang-1, PDGFB and MMP2 in HMEC-1 cells, indicating that Ang-1, PDGFB and MMP2 are the pro-angiogenic factors that might be important in the tryptanthrin-mediated inhibition of angiogenesis. The molecular mechanisms by which tryptanthrin cause the down-regulation of pro-angiogenic factors in vascular endothelial cells remain obscure. Since tryptanthrin is known to be an agonist of the aryl hydrocarbon receptor [45], whether it might exert its effects through the aryl hydrocarbon receptor has yet to be determined.

VEGFR2 is the primary receptor in the VEGF signalling pathway that regulates endothelial cell proliferation, migration, tube formation and angiogenesis. Inhibition of VEGFR2 has been proposed as a promising therapeutic strategy for cancer patients [13], [46]. After binding of VEGF to VEGFR2, VEGFR2 would be activated through autophosphorylation at the Tyr1175 site within its intracellular kinase domain and then initiates a series of downstream signal transduction to endothelial cells [8]. To understand the molecular mechanism of the tryptanthrin-mediated anti-angiogenic effect, the ability of tryptanthrin to inhibit the activation of VEGFR2 was examined. Our findings revealed that tryptanthrin effectively reduced the VEGF-induced Tyr1175 phosphorylation of VEGFR2 to the control level.

Multiple VEGFR2 downstream signalling mediators such as ERK, PI3K-AKT, JNK, and p38 MAPK are known to be involved in regulation of endothelial cell proliferation, survival and migration [9]–[12]. In this study, we found that the phosphorylation of ERK1/2 was significantly inhibited by tryptanthrin. On the other hand, tryptanthrin had little, if any, effects on AKT, JNK and p38 phosphorylation. Our findings are in line with Yue et al. [47] who showed that RA-V, a cyclopeptide isolated from an anti-tumour herb *Rubia yunnanensis*, exhibited its anti-angiogenic activities in HUVEC and HMEC-1 endothelial cells via suppression of the of the ERK1/2 signalling pathway. Similarly, pristimerin, a triterpenoid isolated from *Celastrus* and *Maytenus* spp., could potently inhibit angiogenesis by suppressing the VEGF-induced phosphorylation of VEGFR2 and the activities of AKT, ERK1/2, mTOR, and ribosomal protein S6 kinase [20].

To further understand how tryptanthrin interacted with VEGFR2 to exert its anti-angiogenic effects, the structure-based interaction between tryptanthrin and VEGFR2 was analyzed by molecular docking simulation which is a tool frequently used for evaluating the complex formation of small ligands with large biomolecules. Activation of VEGFR2 kinase is known to be an ATP-consuming process. The ATP-binding site of VEGFR2 is located between the N-terminal lobe and C-terminal lobe within the catalytic domain. Many kinase inhibitors act as ATP minetics and compete with the cellular ATP for the binding with the ATP-binding site and subsequently suppressing the receptor autophosphorylation [48], [49]. Our present study using molecular docking showed that tryptanthrin could bind to the ATP-binding site of VEGFR2 with lower binding energy than ATP, though the actual binding of tryptanthrin to VEGFR2 has yet to be verified experimentally. Collectively, our preliminary results indicate that the binding of tryptanthrin to the ATP-binding pocket and the subsequent inhibition of VEGFR2 phosphorylation and ERK activation might explain the anti-angiogenic action of tryptanthrin.

In conclusion, our findings indicated that tryptanthrin inhibited angiogenesis both *in vitro* and *in vivo* and it might exert its anti-angiogenic effects via blocking of the VEGF/VEGFR2/ERK1/2 signalling axis in human microvascular endothelial cells. Therefore, tryptanthrin can be exploited as a potential therapeutic agent for the treatment of angiogenesis-related diseases.
